# Anterior spinal artery syndrome after double valve replacement and coronary artery bypass surgery

**DOI:** 10.1002/ccr3.928

**Published:** 2017-04-01

**Authors:** Ashwin Subramaniam, Adrian Pick, Ravi Tiruvoipati

**Affiliations:** ^1^ Department of Intensive CareFrankston Hospital and Peninsula Private HospitalFrankstonVictoriaAustralia; ^2^ Monash UniversityMelbourneVictoriaAustralia; ^3^ Peninsula Private HospitalFrankstonVictoriaAustralia

**Keywords:** Anterior spinal artery syndrome, complication of open‐heart surgery, double valve surgery, hemodynamic instability after cardiac surgery, paraplegia

## Abstract

Spinal infarction is a rare and devastating complication of open‐heart surgery, especially in the context of perioperative hemodynamic instability in patients requiring high dose of inotropes and vasoconstrictors. Our report highlights that spinal infarction can occur in such circumstances following a valve replacement surgery.

## Introduction

Paraplegia following any surgery is a rare, yet debilitating complication that is poorly understood. Although the incidence of paraplegia following aortic surgery (such as aortic root replacement or repair of dissection) can be as high as 11% 1, paraplegia related to cardiac surgery is extremely rare. We present an 81‐year‐old lady with known critical aortic stenosis, who underwent double valve surgery and developed anterior spinal artery syndrome postoperatively.

## Case Report

A 81‐year‐old lady who has known critical aortic stenosis (AS) with left ventricular dysfunction presented to the hospital with ischemic chest pain and worsening exertional dyspnea. She was an ex‐smoker but had active history of COPD and depression. There was no history of stroke or peripheral vascular disease.

While awaiting an angiogram, she had brief periods (lasting <30 sec each) of altered speech, confusion, and seizure‐like activity without any neurological or postictal sequelae. CT brain and carotid Doppler ultrasound ruled out a cerebrovascular accident. Echocardiogram confirmed critical AS (mean gradient of 74 mmHg) with moderate mitral regurgitation and pulmonary hypertension (PA systolic 51 mmHg). The LV function was moderately impaired. Her ongoing hypotension (70–85 mmHg systolic BP) necessitated her transfer to intensive care unit (ICU) for hemodynamic optimization prior to her scheduled cardiac surgery. In ICU, her BP improved to around 90/50 mmHg (MAP between 59 and 74 mmHg), with intravenous fluid resuscitation without the need for vasopressor support. She had arterial and central venous cannulation prior to cardiac surgery. Her neurological examination prior to surgery was essentially normal.

The operation was undertaken on cardiopulmonary bypass (CPB) with general anesthesia with no epidural analgesia. CPB cannulation was established uneventfully in the routine fashion, by cannulation of the ascending aorta for arterial return and 2‐stage right atrial cannulation for venous drainage. The aorta was cross‐clamped and heart arrested with antegrade followed by retrograde tepid blood cardioplegia. She had mitral annuloplasty and porcine bioprosthetic aortic valve replacement. Uncomplicated CABG was performed with a single right long saphenous vein graft to the mid‐right coronary artery. Following cross‐clamp release, heart required single DC (10J) shock prior to running stable sinus rhythm. CPB was then slowly discontinued. Her blood pressure was maintained with 0.04 mcg/kg/min of adrenaline and 0.1 mcg/kg/min. The total CPB time was 1:38:16 and cross‐clamp time was 1:25:29. Patient required 4 mg of metaraminol while on CPB.

Upon return to ICU, there was profound hemodynamic instability and low cardiac output with high vasopressor and inotropic requirements [noradrenaline as high as 50 mcg/min (1 mcg/kg/min) and adrenaline 0.2 mcg/kg/min; cardiac index of 1.6 L/min/M^2^, SVR 1415 dynes/sec/cm^5^]. The cardiac output subsequently improved (CI 2.6 L/min/M^2^, SVR 986 dynes/sec/cm^5^) with increase in inotropic support (adrenaline to 0.4 mcg/kg/min). Urgent hemofiltration was initiated for worsening metabolic acidosis and anuria. She also had hyperlactatemia with serum lactate of 10 mmol/L, which was multifactorial, probably a combination of hypotension, poor perfusion, adrenaline use, and liver dysfunction. Immediate bedside transthoracic echocardiogram revealed no aortic or mitral regurgitation with improved LV function and ruled out pericardial effusion. She also had elevated transaminase levels, suggestive of ischemic hepatitis. Given the hemodynamic instability, the patient was deeply sedated and intermittently paralyzed making a thorough neurological examination impossible.

**Figure 1:**
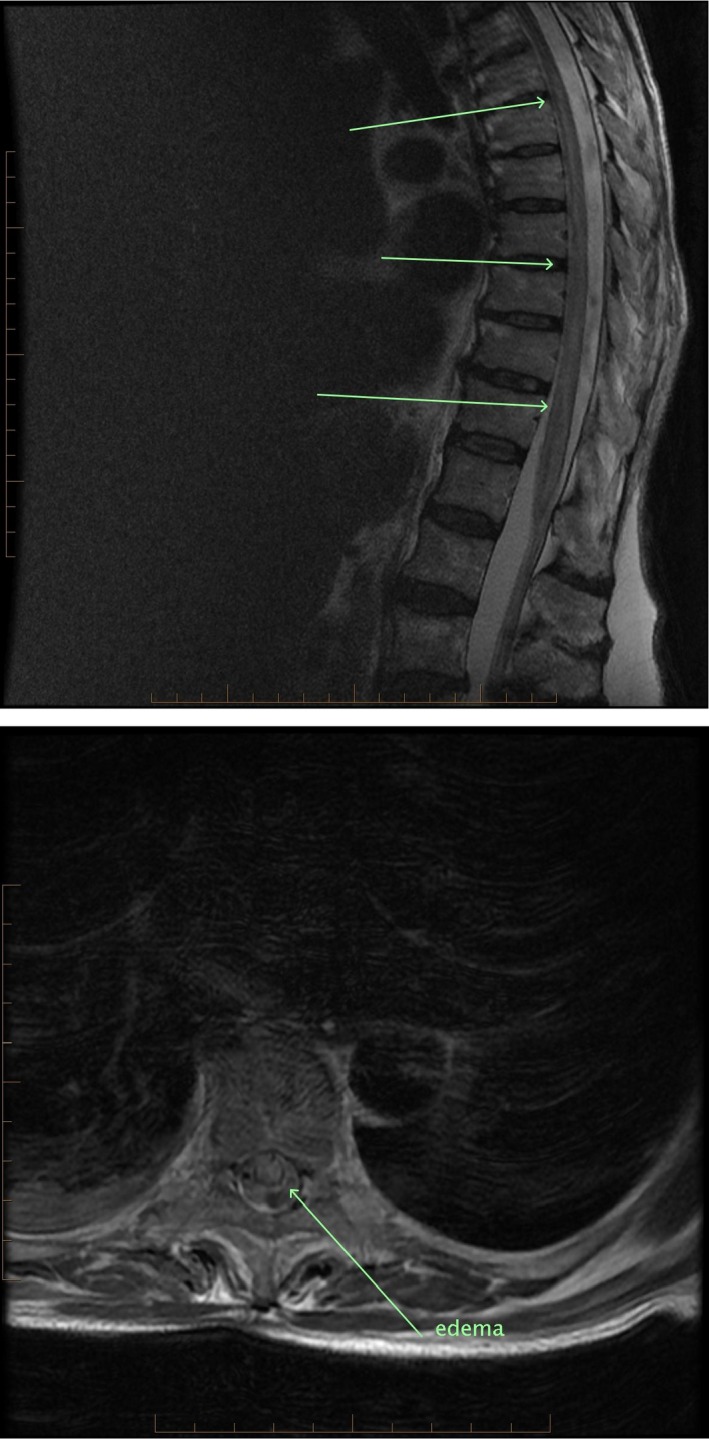
MRI spine showing the extent of involvement from T 6–7 level to conus (image above) and central involvement with thin rim of peripheral sparing of the spinal cord consistent with ASA syndrome.

**Figure 2:**
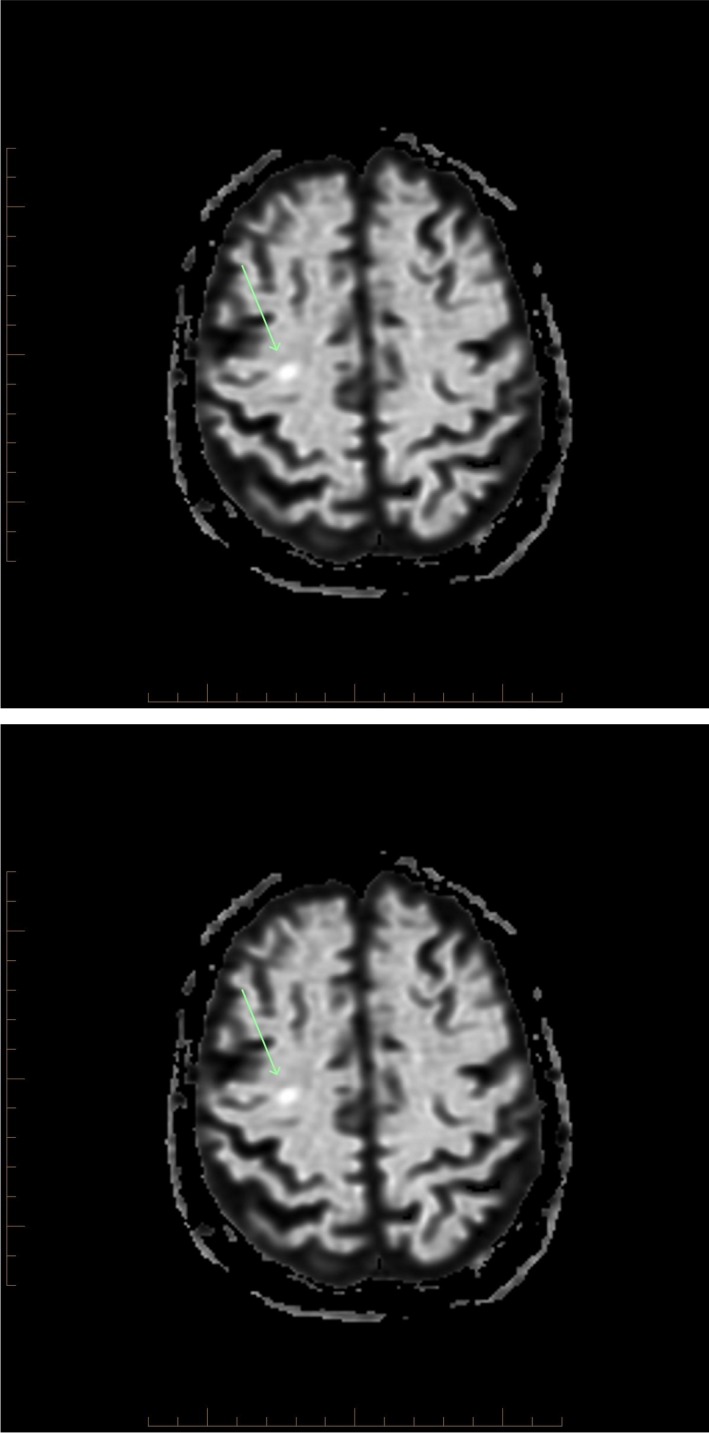
Small acute strokes in subcortical bilateral cerebral hemispheres involving left parieto‐occipital junction corona radiate (Image below) and superior aspect of the central right corona radiate (image above).

The next day, she started to improve with adrenaline weaned off and noradrenaline stabilized between 0.2 mcg/kg/min and 0.36 mcg/kg/min. She started obeying commands moving her upper limbs but not the legs. Neurological examination revealed no motor power in all lower limbs muscle groups, a sensory level at T6‐T7, light touch, and proprioception preserved; however, pain and temperature sensations were absent. There were no bowel or bladder problems. Urgent CT brain was essentially normal; CT spine revealed chronic degenerative changes, but was unable to exclude ischemic spinal injury. Patient was extubated on day‐2. Subsequent MRI spine showed abnormal signals beginning at T6‐7, and extending down to the conus with the cord expanded centrally, the signal was abnormal with a rim of normal periphery all suggestive of anterior spinal artery (ASA) ischemic syndrome (Fig. 1). There was no evidence of aortic dissection or hematoma. MRI brain interestingly revealed two small subcortical acute ischemic infarcts in bilateral cerebral hemispheres, most likely representing embolic phenomena (Fig. 2).

The patient was provided supportive care including hemodynamic optimization and renal replacement therapy (RRT) to assist in her recovery. She required RRT for a further 2 weeks. Her acute kidney injury improved subsequently and was discharged to the ward. She was transferred for rehabilitation after 3 weeks. She underwent intense rehabilitation for 6 weeks and was subsequently discharged home. At 1 year follow‐up, her kidney function had normalized. There has been no improvement in lower limb motor power.

## Discussion

Anterior spinal artery syndrome, most common clinical presentation of a spinal cord infarction, typically presents as loss of motor function and pain/temperature sensation, with relative sparing of proprioception and vibratory sense below the level of the lesion 1, 2. The acute stages are characterized by flaccidity and loss of deep tendon reflexes; spasticity and hyperreflexia develop over ensuing days and weeks. Autonomic dysfunction, sexual dysfunction, and/or bowel and bladder dysfunction may be present 3. Our patient had most features consistent with ASA syndrome.

Prolonged aortic cross‐clamp, low perfusion pressure to the spinal cord, and micro‐emboli 4 to the spinal cord are all proposed causes of spinal infarction 5. It is hard to determine the actual pathophysiological process of our patient's spinal cord dysfunction. Our patient was an ex‐smoker, but did not have any documented history of peripheral vascular disease. She had critical AS with progressive worsening symptoms of presyncopal episodes and exertional dyspnea at presentation. She was also very symptomatic with her hypotension. All these factors put her at a very risk for low flow state. She was hypotensive intra‐operatively as well, requiring vasopressor support. Aortic cross‐clamp time of >60 min is an independent risk factor for low cardiac output 5. Postoperatively, she was in profound cardiogenic shock with multiorgan failure all due to systemic hypotension and low perfusion state. It appears that profound hypotension and requirement of high‐dose vasopressors were the most plausible explanation for her to develop ASA syndrome. It is unclear when she developed the paraplegia, but could have most likely happened intra‐operatively or in the immediate postoperative phase.

Studies have shown that transcatheter aortic valve replacement (TAVR) has shown better survival and quality‐of‐life improvements in clinically inoperable patients with critical AS 6. However, this patient required a double valve surgery and bypass grafting, and hence, TAVR was not an option.

A number of therapies are available to optimize spinal cord perfusion and minimize edema including systemic corticosteroids, permissive hypertension, and diuresis. Drainage of cerebrospinal fluid has been shown to be effective in reversing neurological dysfunction 7. Such treatments have not been studied in spinal infarcts after CABG 4.

Unfortunately, there are no definitive treatments, once the ASA syndrome diagnosis is determined. The mortality rate is approximately 20%, with 50% of individuals not having any recovery of symptoms 8.

It does not appear possible prospectively to identify patients who are likely to have this serious complication. However, patients with symptomatic critical AS should be considered high risk with a likely possibility of spinal infarction. Care must be taken to optimize blood pressure and avoid significant periods of hypotension.

## Conclusion

The exact cause of ASA syndrome in our case is not clear; we believe it is probably due to profound systemic hypotension, high‐dose vasoconstrictors, and/or micro‐embolic phenomenon during perioperative period. While preoperative identification of patients who could develop ASA syndrome is difficult, avoidance of hypotension may reduce the risk of ASA syndrome.

## Authorship

AS: Intensive Care Specialist who cared for the patient in Intensive Care. Write up and review of manuscript and corresponding author. AP: Cardiac surgeon who operated on the patient. Helped with the review of the manuscript. RT: Intensive Care Specialist and Director of ICU, who cared for the patient in Intensive Care. Write up and review of manuscript.

## Conflict of Interest

None declared.
